# Association of hearing loss and tinnitus symptoms with health‐related quality of life among long‐term oropharyngeal cancer survivors

**DOI:** 10.1002/cam4.4931

**Published:** 2022-06-13

**Authors:** Puja Aggarwal, Marc‐Elie Nader, Paul W. Gidley, Raj Pratihar, Shirin Jivani, Adam S. Garden, Frank E. Mott, Ryan P. Goepfert, Christopher Wallace Ogboe, Camille Charles, Clifton D. Fuller, Stephen Y. Lai, G. Brandon Gunn, Erich M. Sturgis, Ehab Y. Hanna, Katherine A. Hutcheson, Sanjay Shete

**Affiliations:** ^1^ Department of Epidemiology The University of Texas MD Anderson Cancer Center Houston Texas USA; ^2^ Department of Head and Neck Surgery The University of Texas MD Anderson Cancer Center Houston Texas USA; ^3^ Department of Radiation Oncology The University of Texas MD Anderson Cancer Center Houston Texas USA; ^4^ Department of Thoracic Head and Neck Medical Oncology The University of Texas MD Anderson Cancer Center Houston Texas USA; ^5^ Department of Otolaryngology‐Head and Neck Surgery Baylor College of Medicine Houston Texas USA; ^6^ Department of Biostatistics The University of Texas MD Anderson Cancer Center Houston Texas United States; ^7^ Division of Cancer Prevention and Population Sciences The University of Texas MD Anderson Cancer Center Houston Texas USA

**Keywords:** hearing loss, oropharyngeal cancer, ototoxicity, survivorship, tinnitus

## Abstract

**Background:**

This study investigated the association of hearing loss and tinnitus with overall health‐related quality of life (HRQoL) among long‐term oropharyngeal cancer (OPC) survivors.

**Methods:**

This study included OPC survivors treated between 2000 and 2013 and surveyed from September 2015 to July 2016. Hearing loss and tinnitus were measured by asking survivors to rate their “difficulty with hearing loss and/or ringing in the ears” from 0 (not present) to 10 (as bad as you can imagine). Hearing loss and tinnitus scores were categorized as follows: 0 for none, 1–4 for mild, and 5–10 for moderate to severe. The primary outcome was the mean score of MD nderson Symptom Inventory Head & Neck module interference component as a HRQoL surrogate dichotomized as follows: 0 to 4 for none to mild and 5 to 10 for moderate to severe interference.

**Results:**

Among 880 OPC survivors, 35.6% (314), reported none, 39.3% (347) reported mild, and 25.1% (221) reported moderate to severe hearing loss and tinnitus. On multivariable analysis, mild (OR, 5.83; 95% CI; 1.48–22.88; *p* = 0.012) and moderate (OR, 30.01; 95% CI; 7.96–113.10; *p* < 0.001) hearing loss and tinnitus were associated with higher odds of reporting moderate to severe symptom interference scores in comparison to no hearing loss and tinnitus. This association of hearing dysfunction was consistent with all domains of HRQoL.

**Conclusions:**

Our findings provide preliminary evidence to support the need for continued audiological evaluations and surveillance to detect hearing dysfunction, to allow for early management and to alleviate the long‐term impact on QoL.

## Graphical abstract

Hearing loss and tinnitus are important treatment‐associated toxicities in head and neck cancer patients and can contribute to severe decline in health‐related quality of life (HRQoL). In the current study, the authors identified an association between hearing loss and tinnitus with worse symptom distress and HRQoL among oropharyngeal cancer (OPC) survivors and this association was consistent across all domains of HRQoL. Findings suggest that preservation of audiological function to enhance HRQoL is vital among growing numbers of OPC survivors.

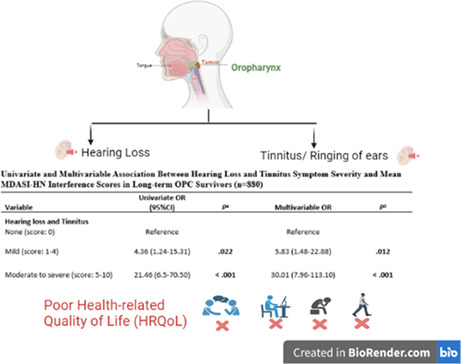

## 1 INTRODUCTION

Advances in treatments and excellent prognosis in patients with human pappillomavirus (HPV)‐associated OPC have contributed to a rapidly growing population of OPC survivors. These survivors are expected to resume normal daily life activities after treatment completion but are at risk of developing toxicities which can have an adverse impact on quality of life (QoL).^1^, ^2^, ^3^, ^4^ Concurrent chemoradiation (CRT) with high‐dose cisplatin is commonly the treatment of choice.^5^, ^6^ This treatment is highly effective, but both radiotherapy and cisplatin‐based CRT in head and neck cancer (HNC) patients can contribute to hearing loss and ototoxicity.^7^ Ototoxicity refers to auditory system dysfunction inclusive of cochlear disorders such as hearing loss and tinnitus; vestibular disorders such as imbalance, vertigo, and dizziness; and neural injury, among others.^8^ Hearing loss can contribute to a constellation of functional deficits in daily life activities, including communication problems with impaired speech perception, mental health problems, psychosocial impairment, cognitive problems such as dementia, low self‐esteem, diminished physical function, and problems with work and employment.^8^, ^9^ In addition, hearing loss attributable to platinum‐based chemotherapy regimens is dose‐dependent and progressive,^8^ and radiotherapy‐associated chronic hearing loss has a long latency period after treatment completion.^8^, ^10^ Overall, hearing loss can contribute to lifelong debilitating consequences for patients and their families, with increased costs in millions of dollars due to decline in work productivity, increased healthcare expenditure, and poor health‐related quality of life (HRQoL).^11^, ^12^

Irradiation fields for head and neck tumors often include the auditory system with the cochlea and the temporal bone, and contribute to the radiotherapy‐associated external, middle, and internal ear injury.^10^, ^13^ Acute effects of radiotherapy include inflammation or edema of the external and middle ear, which can result in conductive hearing loss that may be transient.^14^ However, delayed radiotherapy‐associated injury of the inner ear, including the cochlea and the acoustic nerve, results in permanent sensorineural hearing loss (SNHL).^14^ Platinum‐based chemotherapy can also contribute to injury to the cochlea or retro‐cochlear region including the vestibulocochlear nerve, and other components of the central auditory system,^7^, ^14^, ^15^ resulting in bilateral, progressive, permanent, and high‐frequency SNHL.^14^ Theunissen et al, in a comprehensive review of 21 studies in HNC patients, reported an incidence of SNHL of 0% to 43% immediately following radiotherapy and 17% to 88% in immediately following CRT.^14^ This wide variation in incidence rates may be due to differences in study design, patient selection, treatment, follow‐up duration, and definitions of SNHL among the included studies.^14^ In addition, the risk of worse SNHL was associated with higher radiotherapy dose to cochlea,^14^ CRT with cisplatin,^14^ increase in cumulative cisplatin dose,^14^ increasing age,^14^ longer post‐treatment follow‐up,^14^ suboptimal pretreatment hearing status,^14^ and post‐radiotherapy otitis media.^10^

OPC Treatment can also contribute to tinnitus or ringing in the ears.^8^ Tinnitus may or may not co‐occur with hearing loss; however, patients who report hearing loss are more likely to report tinnitus as well.^8^ Arora et al previously reported that 10.7% of HNC patients who received cisplatin reported tinnitus.^16^ Tinnitus can lead to problems with concentration, sleep, and relationships; impede work; and cause psychosocial dysfunction.^8^

Impairment and interference in daily life activities due to hearing loss and tinnitus can contribute to severe decline in HRQoL, which encompasses functional, physical, psychological, and social domains of health and well‐being. To understand the disease burden due to hearing loss and tinnitus, it is critical to evaluate the association of hearing loss and tinnitus with HRQoL. However, to our knowledge, the association of hearing loss and tinnitus with impairment in daily life activities and HRQoL has not been comprehensively investigated in OPC survivorship. Therefore, the primary objective of this study was to investigate the association of hearing loss and tinnitus symptoms with the severity of cancer treatment–related symptom distress, impairment of daily function, and overall HRQoL among long‐term OPC survivors. Secondary objectives were to investigate the association of hearing loss and tinnitus symptoms with individual domains of HRQoL. We hypothesized that hearing loss and tinnitus would be associated with impairment of daily function, diminished overall HRQoL, and interference in individual domains of HRQoL.

## 2 METHODS

### 2.1 Study population/patient eligibility

A cross‐sectional cancer survivorship survey was administered to 1740 OPC survivors from September 9, 2015, to July 7, 2016. All eligible study participants were adults (age at diagnosis ≥18 years); received curative OPC treatment at The University of Texas MD Anderson Cancer Center during January 1, 2000, to December 31, 2013; and had completed at least 1 year of post‐treatment follow‐up. This study protocol (PA11‐0936) was approved by the MD Anderson Cancer Center institutional review board and included a consent statement on the survey cover letter as informed consent from study participants.

A total of 972 OPC survivors responded (56% response rate), of which 65 survivors with recurrent disease, distant metastasis, and second primary tumors of the head and neck, central nervous system, and thorax were excluded. In addition, 25 patients were excluded for not responding to the hearing loss and tinnitus question on the survey, and two more patients were excluded due to missing the primary outcome (MDASI‐HN symptom interference). In total, 880 OPC survivors were included in the analyses. Additional details of survey administration are available.^17^, ^18^

### 2.2 Survey instrument

The survivorship survey included the MD Anderson Symptom Inventory Head and Neck Cancer Module (MDASI‐HN), a validated multi‐symptom patient‐reported outcomes assessment instrument that evaluates the severity of symptoms and subsequent symptom distress.^19^ The MDASI‐HN assessment includes thirteen core symptoms common across all malignancies/cancers, nine head–neck specific symptoms, and six interference items that evaluate impact on daily life activities and function.^19^ After face validity testing with cognitive debriefing in a pilot project with 50 oropharyngeal cancer survivors, hearing loss and tinnitus were included among three survey‐specific special interest symptom items as an adjunct to the standard MDASI‐HN. The symptom items are rated from 0 (not present) to 10 (as bad as you can imagine), and the mean symptom score represents symptom severity.^19^ The individual interference items are also rated on a scale from 0 (did not interfere) to 10 (interfered completely), and the mean interference score represents symptom distress.^19^ The MDASI‐HN mean subscale scores have internal consistency (Cronbach *α* = 0.72–0.92).^19^

### 2.3 Primary and secondary outcomes

The primary outcome variable for this study was the mean of the interference component of the MDASI‐HN. Mean interference scores were dichotomized per previously defined thresholds: 0 to 4, defined as none to mild interference, and 5 to 10, defined as moderate to severe interference.^17^, ^18^, ^20^, ^21^, ^22^ The mean interference has been reported to be a surrogate for symptom distress, impairment of daily function, and overall HRQoL.^19^, ^23^, ^24^

Six secondary outcomes were the individual interference items: General activity, walking, work, mood, relationships with other people, and enjoyment of life. Individual interference scores were also categorized as 0 to 4 for none to mild interference and 5 to 10 for moderate to severe interference.^17^, ^18^, ^22^, ^23^, ^24^ These secondary outcomes were evaluated to fully explore the association of hearing loss and tinnitus with physical and psychological sub‐domains of HRQoL.

### 2.4 Primary exposure

The hearing loss and tinnitus variable was the primary exposure of interest for our study. Hearing loss and tinnitus was assessed by asking: “How severe are your symptoms? People with cancer frequently have symptoms that are caused by their disease or their treatment. We ask you to rate how severe the symptoms have been in the last 24 hours.” The respondents were then asked to rate their “difficulty with hearing loss and/or ringing of the ears at its worst” on a scale of 0 (not present) to 10 (as bad as you can imagine). As per previous symptom literature regarding symptom thresholds, hearing loss and tinnitus scores were categorized as follows; 0 for none, 1–4 for mild, and 5–10 for moderate to severe hearing loss and tinnitus.^17^, ^18^, ^20^, ^21^, ^22^

### 2.5 Clinical and sociodemographic covariates

Our analyses adjusted for potential clinical and sociodemographic covariates. Information on these covariates were extracted from the electronic medical records of study participants as listed in Table 1 included: Age at diagnosis, gender, race/ethnicity, educational level, smoking status, and HPV and p16 status. Clinical and treatment data abstracted included: Primary OPC tumor subsite; tumor and nodal classifications (American Joint Committee on Cancer version VII); treatment modality; radiotherapy dose, modality, and fractionation; surgery, neck dissection, chemotherapy, and concurrent cisplatin therapy. The difference between the age at the time of the survey and the age at the time of OPC diagnosis was used to compute survival time.

**TABLE 1:** Oropharyngeal cancer patient characteristics (*N* = 880) and distribution by clinical and sociodemographic variables and MD Anderson symptom inventory mean interference scores

Variable	Total patients, No. (%) (N = 880)	Mean interference none to mild, (score:0–4), No. (%) (N = 825, 93.7%)	Mean interference moderate to severe (score: 5–10), No. (%) (N = 55, 6.3%)	*p* ^a^
Hearing loss and Tinnitus				<0.001^a^
None (score: 0)	313 (35.6)	310 (99.0)	3 (1.0)	
Mild (score: 1–4)	346 (39.3)	332 (96.0)	14 (4.0)	
Moderate to severe (score: 5–10)	221 (25.1)	183 (82.8)	38 (17.2)	
Age at diagnosis, median (range, IQR), y	56 (32–84, 34–82)	56 (32–84, 51–63)	55 (41–82, 51–62)	0.646
Survival time, median (range, IQR), y	7 (1–16, 2–16)	6 (1–16, 4–10)	7 (2–15, 4–10)	0.257
Radiation dose, median (range, IQR), Gy	70 (40–72.6, 60–72)	70 (57–72.6, 66–70)	70 (40–72, 66–70)	0.660
Sex				0.019^a^
Male	745 (84.7)	705 (94.6)	40 (5.4)	
Female	135 (15.3)	120 (88.9)	15 (11.1)	
Education				0.848
High school or less	162 (18.4)	152 (93.8)	10 (6.2)	
Greater than high school	636 (72.3)	597 (93.9)	39 (6.1)	
Missing	82 (9.3)	76 (92.7)	6 (7.3)	
Race/ethnicity				0.227
Non‐Hispanic White	813 (92.4)	765 (94.1)	48 (5.9)	
Non‐Hispanic Black	16 (1.8)	14 (87.5)	2 (12.5)	
Hispanic	34 (3.9)	30 (88.2)	4 (11.8)	
Other	8 (0.9)	8 (100.0)	0 (0.0)	
Missing	9 (1.0)	8 (88.9)	1 (11.1)	
Primary tumor site				0.707
Tonsil	410 (46.6)	387 (94.4)	23 (5.6)	
Base of tongue + GPS	439 (49.9)	409 (93.2)	30 (6.8)	
Other	31 (3.5)	29 (93.6)	2 (6.4)	
T classification				0.146
T1 + T2	665 (75.6)	628 (94.4)	37 (5.6)	
T3 + T4	215 (24.4)	197 (91.6)	18 (8.4)	
N classification				0.294
N0 + N1 + N2a + N2b	706 (80.2)	665 (94.2)	41 (5.8)	
N2c + N3	174 (19.8)	160 (92.0)	14 (8.0)	
HPV status				
Negative	56 (6.4)	54 (96.4)	2 (3.6)	0.641
Positive	427 (48.5)	402 (94.2)	25 (5.8)	
Unknown	397 (45.1)	369 (93.0)	28 (7.0)	
Cigarette smoking				0.036^a^
Never	412 (46.8)	390 (94.7)	22 (5.3)	
Former smoker at diagnosis	331 (37.6)	312 (94.3)	19 (5.7)	
Quit smoking subsequent to diagnosis	90 (10.2)	84 (93.3)	6 (6.7)	
Current smoker at survey	35 (4.0)	28 (80.0)	7 (20.0)	
Do not know	12 (1.4)	11 (91.7)	1 (8.3)	
Treatment group				0.174
Single modality	271 (30.8)	259 (95.6)	12 (4.4)	
Multimodality	609 (69.2)	566 (92.9)	43 (7.1)	
Surgery				1.000
No	857 (97.4)	803 (93.7)	54 (6.3)	
Yes – robotic	18 (2.0)	17 (94.4)	1 (5.6)	
Yes – open	5 (0.6)	5 (100.0)	0 (0.0)	
Neck dissection				1.000
No	659 (74.9)	618 (93.8)	41 (6.2)	
Yes	221 (25.1)	207 (93.7)	14 (6.3)	
RT				1.000
No	8 (0.9)	8 (100.0)	0 (0.0)	
Yes	872 (99.1)	817 (93.7)	55 (6.3)	
RT schedule				0.625
Standard fractionation	777 (88.3)	730 (94.0)	47 (6.0)	
Accelerated	95 (10.8)	87 (91.6)	8 (8.4)	
Missing/no RT	8 (0.9)	8 (100.0)	0 (0.0)	
RT type				0.190
3D conformal	49 (5.6)	43 (87.8)	6 (12.2)	
MRT Bilateral (SF + WF + VMAT) + Proton	725 (82.4)	679 (93.7)	46 (6.3)	
IMRT Ipsilateral	98 (11.1)	95 (96.9)	3 (3.1)	
Missing/no RT	8 (0.9)	8 (100.0)	0 (0.0)	
Chemotherapy				0.134
No	278 (31.6)	266 (95.7)	12 (4.3)	
Yes	602 (68.4)	559 (92.9)	43 (7.1)	
Concurrent cisplatin therapy				0.524
None	644 (73.2)	607 (94.2)	37 (5.8)	
Concurrent high‐dose cisplatin	95 (10.8)	89 (93.7)	6 (6.3)	
Concurrent low‐dose cisplatin	122 (13.8)	111 (91.0)	11 (9.0)	
Other concurrent cisplatin	19 (2.2)	18 (94.7)	1 (5.3)	

Abbreviations: 3D‐CRT, three‐dimensional conformal radiotherapy; GPS, glossopharyngeal sulcus; IMRT‐SF, intensity modulated radiotherapy split‐field technique; IMRT‐WF, intensity modulated radiotherapy whole‐field technique; IQR, interquartile range; RT, radiotherapy; T, tumor; VMAT, volumetric‐modulated arc therapy.

^a^ Statistical significance was defined as *p* ≤ 0.05.

### 2.6 Statistical analysis

Descriptive analysis was conducted to summarize the mean interference categories across demographic and clinical variables. Univariate logistic regression and multivariable logistic regression were conducted to investigate unadjusted and adjusted associations, respectively, between hearing loss and tinnitus and interference scores. Similar analyses were conducted to investigate associations between hearing loss and tinnitus and secondary outcomes. All multivariable logistic regression models controlled for age at diagnosis, radiotherapy dose, survival time, sex, race, education, tumor subsite, T‐classification, N‐classification, HPV status, cigarette smoking, treatment modality, surgery, neck dissection, radiotherapy schedule, radiotherapy type, chemotherapy, and concurrent cisplatin therapy. Univariate and multivariable odds ratios and their corresponding 95% CIs were computed. A variance inflation factor greater than 10 was the criterion used to establish any multicollinearity. Two‐sided *p* ≤ 0.05 was considered statistically significant. Analysis was performed using STATA 14.0 (StataCorp).

## 3 RESULTS

### 3.1 Sample characteristics

Among 880 OPC survivors the median age at diagnosis was 56 years (range, 32–84), the median survival duration at the time of survey was 7 years (range, 1–16 years), and the median total RT dose was 70 Gy (range, 40–72.6 Gy). Of these 84.7% (745/880) were males, 92.4% (813/880) were non‐Hispanic white, and 72.3% (636/880) had greater than high school education. The study population's clinical and sociodemographic characteristics, stratified by mean interference scores, are summarized in Table 1. Of 880 study participants, 313 (35.6%) reported no hearing loss and tinnitus, 346 (39.3%) reported mild hearing loss and tinnitus, and 221 (25.1%) reported moderate to severe hearing loss and tinnitus. Among patients with moderate to severe hearing loss and tinnitus, 38 (17.2%) reported moderate to severe mean interference scores.

### 3.2 Primary outcome: Association between hearing loss and tinnitus symptom severity and mean interference

Table 2 summarizes the univariate and multivariable regression for hearing loss and tinnitus symptom severity with interference severity categories. On univariate analysis, none to mild hearing loss and tinnitus, moderate to severe hearing loss and tinnitus, female sex, and current smoking at time of survey were associated with higher odds of reporting moderate to severe interference scores. Intensity‐modulated radiotherapy (IMRT) ipsilateral or unilateral irradiation techniques which aim to limit treatment to the primary tumor and the neck on the same side was associated with lower odds of reporting moderate to severe interference scores. On multivariable analysis controlled for clinico‐demographic variables, mild hearing loss and tinnitus (OR, 5.83; 95% CI, 1.48–22.88; *p* = 0.012) and moderate to severe hearing loss and tinnitus (OR, 30.01; 95% CI, 7.96–113.10; *p* < 0.001) were significantly associated with higher odds of reporting moderate to severe mean interference scores compared with no hearing loss and tinnitus.

**TABLE 2:** Univariate and multivariable association between hearing loss and tinnitus symptom severity and mean MDASI‐HN interference scores in long‐term OPC survivors (*n* = 880)

Variable	Univariate OR	Lower CI	Upper CI	*p* ^a^	Multivariable OR	Lower CI	Upper CI	*p* ^*b*^
Hearing loss and Tinnitus								
None (score: 0)	Reference				Reference			
Mild (score: 1–4)	4.36	1.24	15.31	0.022^a^	5.83	1.48	22.88	0.012^*b*^
Moderate to severe (score: 5–10)	21.46	6.53	70.50	<0.001^a^	30.01	7.96	113.10	<0.001^*b*^
Age at diagnosis	1.00	0.97	1.03	0.828	1.00	0.96	1.04	0.981
Survival time	1.01	0.95	1.09	0.691	0.95	0.82	1.10	0.472
Radiation dose	1.00	0.90	1.12	0.938	0.85	0.75	0.96	0.009^*b*^
Sex								
Male	Reference				Reference			
Female	2.20	1.18	4.11	0.013^a^	3.18	1.51	6.72	0.002^*b*^
Education								
High school or less	Reference				Reference			
Greater than high school	0.99	0.48	2.03	0.985	1.26	0.56	2.87	0.575
Missing	1.20	0.42	3.43	0.733	1.34	0.40	4.46	0.636
Race/ethnicity								
Non‐Hispanic White	Reference				Reference			
Non‐Hispanic Black	2.28	0.50	10.31	0.286	7.39	0.93	58.52	0.058
Hispanic	2.13	0.72	6.28	0.173	1.58	0.46	5.48	0.470
Other	1.00				1.00			
Missing	1.99	0.24	16.26	0.520	1.49	0.13	17.41	0.750
Primary tumor site								
Tonsil	Reference				Reference			
Base of Tongue + GPS	1.23	0.70	2.16	0.462	1.07	0.55	2.08	0.848
Other	1.16	0.26	5.17	0.845	0.73	0.12	4.62	0.743
T classification								
T1 + T2	Reference				Reference			
T3 + T4	1.55	0.86	2.79	0.142	1.27	0.57	2.79	0.559
N classification								
N0 + N1 + N2a + N2b	Reference				Reference			
N2c + N3	1.42	0.76	2.67	0.277	0.93	0.43	2.02	0.851
HPV status								
Negative	Reference				Reference			
Positive	1.68	0.39	7.29	0.489	2.50	0.48	12.98	0.275
Unknown	2.05	0.47	8.85	0.337	2.63	0.50	13.78	0.251
Cigarette smoking								
Never	Reference				Reference			
Former smoker at diagnosis	1.08	0.57	2.03	0.812	1.35	0.67	2.74	0.403
Quit smoking subsequent to diagnosis	1.27	0.50	3.22	0.620	1.50	0.52	4.32	0.447
Current smoker at survey	4.43	1.74	11.27	0.002^a^	3.57	1.18	10.84	0.024^*b*^
Do not know	1.61	0.20	13.05	0.655	2.63	0.29	24.05	0.391
Treatment group								
Single modality	Reference				Reference			
Multimodality	1.64	0.85	3.16	0.140	1.13	0.47	2.71	0.785
Surgery								
No	Reference				Reference			
Yes – robotic	0.87	0.11	6.70	0.897	1.34	0.12	14.64	0.809
Yes – open	NA				NA			
Neck dissection								
No	Reference				Reference			
Yes	1.02	0.54	1.91	0.952	1.20	0.58	2.48	0.626
RT schedule								
Standard fractionation	Reference				Reference			
Accelerated	1.43	0.65	3.12	0.372	2.01	0.63	6.38	0.235
Missing/no RT	NA				NA			
RT type								
3d conformal	Reference				Reference			
IMRT Bilateral (SF + WF + VMAT) + Proton	0.49	0.20	1.20	0.118	0.37	0.09	1.47	0.158
IMRT Ipsilateral	0.23	0.05	0.95	0.042^a^	0.14	0.02	0.97	0.046^b^
Missing/no RT	NA				NA			
Concurrent cisplatin therapy								
None	Reference				Reference			
Concurrent high‐dose cisplatin	1.11	0.45	2.70	0.825	0.84	0.28	2.55	0.762
Concurrent low‐dose cisplatin	1.63	0.81	3.28	0.175	2.11	0.87	5.17	0.101
Other concurrent cisplatin	0.91	0.12	7.02	0.929	0.44	0.04	4.85	0.507

*Note*: Statistical significance was defined as *p* ≤ 0.05.

Abbreviations: 3D‐CRT, three‐dimensional conformal radiotherapy; GPS, glossopharyngeal sulcus; IMRT‐SF, intensity modulated radiotherapy split‐field technique; IMRT‐WF, intensity modulated radiotherapy whole‐field technique; IQR, interquartile range; RT, radiotherapy; T, tumor; VMAT, volumetric‐modulated arc therapy.

^a^ *p* ≤ 0.05 after univariate analysis.

^b^ *p* ≤ 0.05 after multivariable analysis.

### 3.3 Secondary outcomes: Association between hearing loss and tinnitus and individual interference items

Table 3 summarizes the univariate and multivariable regression analyses for hearing loss and tinnitus with individual interference items. For activity‐related domains, on multivariable analysis, moderate to severe hearing loss and tinnitus was associated with higher odds of reporting moderate to severe interference with general activity (OR, 10.84; 95% CI, 5.03–23.36; *p* < 0.001) in comparison to no hearing loss and tinnitus. Also, moderate to severe hearing loss and tinnitus was associated with higher odds of reporting moderate to severe interference with work (OR, 6.03; 95% CI, 3.11–11.71; *p* < 0.001) in comparison to no hearing loss and tinnitus. Further, moderate to severe hearing loss and tinnitus were associated with higher odds of reporting moderate to severe interference with walking (OR, 16.93; 95% CI, 5.80–49.43; *p* < 0.001) in comparison to no hearing loss and tinnitus.

**TABLE 3:** Univariate and multivariable adjusted odds ratios for hearing loss and tinnitus symptom severity and individual MDASI‐HN interference items in long‐term OPC survivors (n = 880)

MDASI‐HN individual interference items	Univariate OR	Lower CI	Upper CI	*p* ^a^	Multivariable OR	Lower CI	Upper CI	*p* ^b^
General activity								
Hearing loss and tinnitus								
None (score: 0)	Reference				Reference			
Mild (score: 1–4)	1.88	0.87	4.08	0.110	1.94	0.86	4.39	0.112
Moderate to severe (score: 5–10)	9.60	4.75	19.39	<0.001^a^	10.84	5.03	23.36	<0.001^b^
Mood								
Hearing loss and tinnitus								
None (score: 0)	Reference				Reference			
Mild (score: 1–4)	2.60	1.14	5.93	0.023 ^a^	2.64	1.11	6.28	0.028^b^
Moderate to severe (score: 5–10)	10.10	4.65	21.93	<0.001^a^	10.79	4.66	25.00	<0.001^b^
Work								
Hearing loss and tinnitus								
None (score: 0)	Reference				Reference			
Mild (score: 1–4)	1.62	0.86	3.05	0.137	1.67	0.85	3.30	0.139
Moderate to severe (score: 5–10)	5.63	3.11	10.18	<0.001^a^	6.03	3.11	11.71	<0.001^b^
Relations with other people								
Hearing loss and tinnitus								
None (score: 0)	Reference				Reference			
Mild (score: 1–4)	2.56	1.06	6.18	0.036^a^	2.56	1.03	6.39	0.043^b^
Moderate to severe (score: 5–10)	9.74	4.27	22.19	<0.001^a^	9.17	3.82	21.99	<0.001^b^
Walking								
Hearing loss and tinnitus								
None (score: 0)	Reference				Reference			
Mild (score: 1–4)	2.21	0.77	6.35	0.140	2.71	0.86	8.57	0.090
Moderate to severe (score: 5–10)	13.33	5.16	34.45	<0.001^a^	16.93	5.80	49.43	<0.001^b^
Enjoyment of life								
Hearing loss and tinnitus								
None (score: 0)	Reference				Reference			
Mild (score: 1–4)	1.33	0.69	2.56	0.401	1.40	0.70	2.81	0.342
Moderate to severe (score: 5–10)	5.21	2.87	9.45	<0.001^a^	5.73	2.96	11.09	<0.001^b^

*Note*: Statistical significance was defined as *p* ≤ 0.05. All multivariable models were controlled for age at diagnosis, radiotherapy dose, survival time, sex, race, education, subsite, T‐classification, N‐classification, HPV status, cigarette smoking status, treatment modality, chemotherapy, concurrent cisplatin therapy, surgery, neck dissection, radiotherapy schedule, and radiotherapy type.

Abbreviations: MDASI‐HN, MD Anderson symptom inventory head and neck cancer module; OR, odds ratio.

^a^ *p* ≤0.05 after univariate analysis.

^b^ *p* ≤0.05 after multivariable analysis.

For psychosocial domains, on multivariable analysis mild hearing loss and tinnitus (OR, 2.64; 95% CI, 1.11–6.28; *p* = 0.028) and moderate to severe hearing loss and tinnitus (OR, 10.79; 95% CI, 4.66–25.00; *p* < 0.001) were significantly associated with higher odds of reporting moderate to severe interference in mood in comparison to no hearing loss and tinnitus. Also, mild hearing loss and tinnitus (OR, 2.56; 95% CI, 1.03–6.39; *p* = 0.043) and moderate to severe hearing loss and tinnitus (OR, 9.17; 95% CI, 3.82–21.99; *p* < 0.001) were significantly associated with higher odds of reporting moderate to severe interference in relations with other people in comparison to no hearing loss and tinnitus. Lastly, moderate to severe hearing loss and tinnitus was associated with higher odds of reporting moderate to severe interference with the enjoyment of life (OR, 5.73; 95% CI, 2.96–11.09; *p* < 0.001) in comparison to no hearing loss and tinnitus.

## 4 DISCUSSION

This large survivorship study is first, to our knowledge, to provide a comprehensive quantitative assessment of the associations between important measures of hearing symptom severity inclusive of overall hearing loss and tinnitus and severity of symptom distress, impairment of daily function, overall HRQoL, and individual functional and psychosocial domains of HRQoL among long‐term OPC survivors. The study results indicate that the overall symptom distress was low in OPC survivors, with 6.3% (55/880) reporting moderate to severe impairment in overall HRQoL. Importantly, 64.4% of the OPC survivors reported mild to severe hearing symptoms, including 25.1% who reported moderate to severe hearing dysfunction, indicating the need for supportive treatment to optimize audiological function in this population. Hearing dysfunction as measured by hearing loss and/or tinnitus symptom severity was associated with worse overall HRQoL and symptom distress in our study. Furthermore, hearing loss and tinnitus symptoms were also associated with all the individual domains of multidimensional HRQoL, including general activity, walking, work, mood, relationships with other people, and enjoyment of life. Effect estimates for the association of hearing dysfunction with overall HRQoL and individual components of HRQoL were significantly large; however, confidence intervals were wider because of the small sample sizes. Our study results raise great concern given the importance of hearing function in all aspects of life.

Our results suggest that OPC survivors who experienced mild hearing loss and tinnitus had 5.8 times and those who experienced moderate to severe hearing loss and tinnitus had 30.0 times greater odds of reporting moderate to severe mean interference scores adjusting for clinical, and demographic covariates compared to survivors with no hearing loss and tinnitus. As mean interference score reflects impairment in multiple domains including functional and psychosocial components, our study findings suggest substantial downstream negative sequelae of loss of hearing function on overall HRQoL.^27^ Our study results are plausible because hearing function is critical for daily life activities related to communication, work‐life, home‐life, social‐life, and overall mental well‐being.^8^ Therefore, hearing loss can contribute to impairment of physical functioning, cognitive function, and psychosocial health and can contribute to decline in HRQoL in long‐term aging OPC survivors.^25^ In a cross‐sectional study among long‐term cancer survivors, Stava et al reported that a higher proportion of HNC survivors with hearing loss (24 of 30, 80%) versus HNC survivors without hearing loss (43 of 141, 30%) reported changes in overall health and well‐being.^26^ Also, a higher proportion of HNC survivors with hearing loss (17%) reported problems with work versus those with normal hearing (5%).^26^ In addition, Arlinger et al reported in a review that hearing loss, if left untreated, can have a detrimental effect on QoL.^27^ In fact, Enoch et al reported hearing function along with balance as among the three most important sensory functions in a general adult study population.^28^ Hearing loss can also contribute to difficulties in comprehension of health‐related communication and act as a barrier to supportive care and counseling of affected individuals.^29^ Therefore, loss of hearing function, especially irreversible SNHL for which there is a lack of effective treatment,^29^ can have long‐standing and devastating physical, functional, cognitive, social, emotional, and financial consequences along with the life of OPC survivors.^30^

In addition to hearing loss and tinnitus, total radiotherapy dose to the primary tumor site, female sex, and current smoking at the time of survey were associated with increased odds of reporting moderate to severe interference scores in multivariable models in our study. Only IMRT ipsilateral regimens were associated with lower odds of reporting moderate to severe interference scores. It has been hypothesized that higher total radiotherapy dose to the primary tumor site can contribute to increased irradiation of adjacent nerves, blood vessels, and structures in the head and neck region and subsequent injury that can lead to increased morbidity, impairment of function, and diminished QoL.^31^ Also, the association of female sex with interference scores in our study is consistent with multiple studies in HNC patients, which reported that female patients are more likely to report worse physical, functional, and emotional problems/impairment and diminished QoL than male patients.^32^, ^33^, ^34^, ^35^ Association of smoking with worse interference scores in our study is also consistent with multiple studies in HNC patients which demonstrate that smoking can lead to worse QoL during and after the completion of radiotherapy.^32^, ^36^, ^37^, ^38^ Lastly, protective association of ipsilateral IMRT with interference scores is also expected because IMRT minimizes radiation exposure to neighboring healthy tissues and critical structures and ipsilateral regimens deliver unilateral radiotherapy, which further maximizes sparing of the critical structures, contributing to less radiotherapy‐associated injury and subsequent treatment‐related morbidity.^39^, ^40^, ^41^

However, our study did not identify other reported associations between age,^42^, ^43^ race,^42^, ^43^ primary tumor site,^43^, ^44^ tumor stage,^43^, ^44^ nodal stage,^45^ treatment modality,^45^ postoperative radiotherapy regimens,^45^ and adjuvant chemotherapy^45^ with QoL in HNC patients. Some potential reasons could be differences in definitions of HRQoL and interference, differences in assessment of HRQoL/interference, use of clinically relevant interference categories, and our focus on OPC versus more diverse HNC tumors studied in other investigations.

Given the importance of hearing function in daily life activities, communication, and function, the results of our secondary analysis revealing the association of hearing loss and tinnitus with all domains of HRQoL including functional components such as general activity, walking, and work and psychosocial components such as mood, relations with other people, and enjoyment of life is illuminating. Hearing loss and tinnitus may also add additional morbidity with vestibular dysfunction causing imbalance, dizziness, and vertigo, although definitive studies on cisplatin‐induced vestibular symptoms are lacking.^8^ Further, platinum‐based chemotherapy can contribute to ototoxicity, neuropathy, and problems with balance.^8^ Therefore, patients with hearing loss may also experience problems with balance and falls and even be fearful of falling and injuring themselves.^8^ These problems can contribute to compromised/diminished physical function with decline in general activity and walking. Studies suggest that hearing dysfunction is correlated with deterioration in physical function in the general population.^46^, ^47^ Carabellese et al also reported decline in self‐sufficiency pertaining to daily life function in individuals with SNHL.^48^ Also, the association of hearing loss with falls and diminished physical function has been reported.^9^

Hearing loss and associated deficits with communication, physical function, cognitive function, and psychosocial impairment can also contribute to impairment of work‐life and contribute to problems with employment.^8^ A study reported that 70% of individuals with hearing loss experienced problems with employment or meeting their work requirements and felt isolated.^49^ Thus, hearing dysfunction can contribute to increased morbidity, burden, and cost due to diminished work productivity.^50^

Hearing loss with inability to comprehend conversation and associated communication problems can have a debilitating impact of psychosocial domains of life, with decline in interpersonal relationships, low self‐esteem, frustration, embarrassment, social isolation/withdrawal, depression, inability to enjoy life, and overall poor HRQoL.^8^, ^51^ A previous study reported that individuals with SNHL were more likely to have depression.^47^ Hearing loss also has been associated with cognitive impairment, dementia,^52^ mental health, and psychosocial problems.^53^ The enjoyment of acoustic interests, such as music, and the challenge of discovering new ways of navigating entertainment and business to correspond with the adjusted level of hearing pose barriers to enjoyment of life and additional burden on the patient QOL. In summary, loss of hearing function has a multidimensional, debilitating, long‐term, and permanent impact on every domain of HRQoL of patients and their families and needs to be addressed.

Our study has some limitations that need to be acknowledged. First, the hearing loss and tinnitus assessment were patient‐reported with a single question without the ability to study the symptoms independent of one another. Another critical limitation is that the hearing outcomes here are exclusively by patient‐report without any objective audiological testing of hearing function. However, patient‐reported assessment in our study allows evaluation of ototoxicity and associated impairment in daily life and function from a patient perspective, leaving the opportunity to build upon these patient‐centered data in future studies incorporating more conventional clinical audiologic and vestibular measurements. Small sample sizes across sub‐groups contributed to wide confidence intervals for effect estimates in our study; however, effect estimates across HRQoL domains showed a consistent detrimental impact of hearing loss. Also, we could not assess the association of hearing loss and tinnitus separately on interference scores because our question combined hearing loss and tinnitus together, and future studies might consider using domain‐specific PROs such as the Tinnitus Handicap Inventory^8^ along with separate assessments for different types of hearing loss. We did not have information on baseline hearing assessments prior to treatment which could have provided further information to differentiate between treatment‐related or tumor‐related hearing loss and their association with overall HRQoL. Furthermore, we did not have information on hearing assessment before treatment. Survival bias could also have an impact on our study results, which we statistically controlled by adjusting for survival time in all analytical models. Lastly, we did not have information on radiation dose to the cochlea which has previously been associated with risk of SNHL in head and neck cancer patients,^5^ therefore, future OPC studies should incorporate information about cochlear dose. However, it has been shown that when mean cochlear doses are below 35 Gy risk of sensorineural hearing loss from radiation is minimal.^54^ Importantly, dose to the cochlea with modern IMRT in OPC patients is far below this threshold. Therefore, inability to correct for radiation dose to the cochlea is unlikely to be a major limitation in the current study, as IMRT was adopted early at our institution.

Our study results can inform future studies and potential development of surveillance strategies and supportive care interventions that can be implemented early to prevent irreversible hearing loss and alleviate the impact of hearing dysfunction in OPC survivors. Management of hearing loss may include the use of hearing aids or assistive hearing devices, middle ear or cochlear implants for sound amplification, and individual psychological counseling regarding other communication strategies.^9^ Long‐term surveillance of hearing problems is crucial and may include conventional pure tone audiometry testing for the diagnosis of different types of hearing loss/dysfunction,^8^ high‐frequency audiograms for early ototoxicity detection,^8^ and audiometric measures for speech comprehension in quiet and noisy environments.^8^, ^9^ Ototoxicity protective agents such as sodium thiosulfate, *N*‐acetylcysteine, and other agents may also be further investigated and considered to protect patients from hearing problems.^5^

Given the substantial burden of diseases due to hearing loss and tinnitus reported in our study, it is of utmost importance that ototoxicity be further investigated in the OPC patient population. Future prospective longitudinal studies investigating ototoxicity need to incorporate objective as well as patient‐reported measures, uniformly define clinically significant hearing loss criteria, and include audiology hearing assessments at baseline, during treatment, and long‐term post‐treatment. Different types of hearing loss, laterality of hearing loss, tinnitus, and balance problems and cognitive impairment, depression, and social isolation due to hearing loss also need to be investigated. Identification of predictors of ototoxicity can also help stratify patient populations at high‐risk of hearing dysfunction for targeted rehabilitation therapy. Furthermore, there is a need for the development of an effective evidence‐based ototoxicity surveillance and management program with uniform definitions of ototoxicity and standard criteria and grading for audiological and associated HRQoL assessments to mitigate some of the downstream impacts of hearing dysfunction on QoL in OPC survivors.

## 5 CONCLUSIONS

Hearing dysfunction is a serious treatment‐related toxicity, and prolonged life with hearing impairment has been shown to be profoundly concerning.^8^, ^28^ Overall, in this large survivorship survey study, 1 of 4 long‐term OPC survivors reported moderate to severe hearing loss and tinnitus, and a sizeable proportion of these (17.2%) reported moderate to severe interference and functional impairment. Our study results need to be validated in future longitudinal studies; nonetheless, they provide preliminary evidence of devastating consequences of hearing loss and tinnitus on functional impairment, overall HRQoL, individual domains of HRQoL and overall well‐being of OPC survivors. Our findings can inform clinicians/ health‐care providers and patients about the disease burden and distress associated with ototoxicity. Our study results also indicate the unmet need for long‐term monitoring of hearing function and rehabilitative efforts despite existing clinical guidelines for surveillance and management of hearing dysfunction. Ototoxicity and its debilitating effects on QoL for both patients and their families need further investigation in the growing number of OPC survivors at risk of long‐term consequences of ototoxicity and associated decline in HRQoL.

## AUTHOR CONTRIBUTIONS

Puja Aggarwal: Substantial contributions to the study conception or design; data management, supervised analysis, and interpretation; drafting manuscript, manuscript revisions; and contributions to the final version of the manuscript. Marc‐Elie Nader, Paul W. Gidley, Raj Pratihar, Shirin Jivani, Adam S. Garden, Frank E. Mott, Ryan P. Goepfert, Christopher Wallace Ogboe, Camille Charles, Clifton D. Fuller, Stephen Y. Lai, G. Brandon Gunn, Erich M. Sturgis, Ehab Y. Hanna: Clinical data interpretation and analytic support; *s*ubstantial contributions to, critical revision of the manuscript for important intellectual content, and contributions to the final version of the manuscript. Katherine A. Hutcheson: Responsible for data collection, substantial contributions to the study conception or design or the acquisition; interpretation of data; critical revision of the manuscript for important intellectual content, and contributions to the final version of the manuscript. Sanjay Shete: Concept and design, interpretation of data, initial draft of the manuscript, study supervision, critical revision of the manuscript for important intellectual content, and contributions to the final version of the manuscript. All authors agreed to be accountable for all aspects of the work in ensuring that questions related to the accuracy or integrity of any part of the work were appropriately investigated and resolved.

## FUNDING INFORMATION

This study was supported by the NIH/NCI under award number 5P30CA016672 (SS), a Cancer Prevention Fellowship supported by the Cancer Prevention and Research Institute of Texas (CPRIT) grant award, RP170259 (to PA; PI: S. Chang and S. Shete), and the Betty B. Marcus Chair in Cancer Prevention (SS). The OPC Survivorship Survey data collection was supported by the Charles and Daneen Stiefel Oropharynx Fund. This study was also supported by the Alvin R. Tarlov and John E. Ware Jr. Post‐Doctoral Award in Patient‐Reported Outcomes for 2020‐2021 (to Puja Aggarwal). Mark‐Ellie Nader reports stock ownership in 3M, Amgen, Cardinal Health, Johnson & Johnson, Medtronic, and Pfizer.

## CONFLICT OF INTEREST

The funding sources had no role in the design and conduct of the study; data collection, management, analysis, and interpretation of the data; preparation, review, or approval of the manuscript; and decision to submit the manuscript for publication.

## ETHICAL APPROVAL STATEMENT

This study (PA11‐0936) was approved by the MD Anderson Cancer Center institutional review board prior to commencing the study.

## Data Availability

The dataset used in this study is not publicly available as it contains patient identifiers. The data supporting the findings of our study can be obtained from the corresponding author.
